# The starvation hormone, fibroblast growth factor-21, extends lifespan in mice

**DOI:** 10.7554/eLife.00065

**Published:** 2012-10-15

**Authors:** Yuan Zhang, Yang Xie, Eric D Berglund, Katie Colbert Coate, Tian Teng He, Takeshi Katafuchi, Guanghua Xiao, Matthew J Potthoff, Wei Wei, Yihong Wan, Ruth T Yu, Ronald M Evans, Steven A Kliewer, David J Mangelsdorf

**Affiliations:** 1 Department of Pharmacology, University of Texas Southwestern Medical Center, Dallas, United States; 2 Department of Clinical Sciences, University of Texas Southwestern Medical Center, Dallas, United States; 3 Hypothalamic Research, University of Texas Southwestern Medical Center, Dallas, United States; 4 Department of Pharmacology, Howard Hughes Medical Institute, University of Texas Southwestern Medical Center, Dallas, United States; 5 Advanced Imaging Research Center, University of Texas Southwestern Medical Center, Dallas, United States; 6 Gene Expression Laboratory, Howard Hughes Medical Institute, Salk Institute for Biological Studies, San Diego, United States; 7 Departments of Molecular Biology and Pharmacology, University of Texas Southwestern Medical Center, Dallas, United States; University of Massachusetts Medical School, United States

**Keywords:** longevity, fibroblast growth factor, growth hormone, liver, caloric restriction, Mouse

## Abstract

Fibroblast growth factor-21 (FGF21) is a hormone secreted by the liver during fasting that elicits diverse aspects of the adaptive starvation response. Among its effects, FGF21 induces hepatic fatty acid oxidation and ketogenesis, increases insulin sensitivity, blocks somatic growth and causes bone loss. Here we show that transgenic overexpression of FGF21 markedly extends lifespan in mice without reducing food intake or affecting markers of NAD+ metabolism or AMP kinase and mTOR signaling. Transcriptomic analysis suggests that FGF21 acts primarily by blunting the growth hormone/insulin-like growth factor-1 signaling pathway in liver. These findings raise the possibility that FGF21 can be used to extend lifespan in other species.

**DOI:** http://dx.doi.org/10.7554/eLife.00065.001

## eLife digest

In 1934, in a famous experiment at Cornell University, it was discovered that laboratory mice could live twice as long as expected if they were fed a low-calorie diet that included enough nutrients to avoid malnutrition. This phenomenon has since been observed in species ranging from worms to primates, but not in humans. Reducing calorie intake leads to longer lives by modifying a number of the biochemical pathways that sense nutrients, including pathways that involve insulin and various other biomolecules. Chemical and genetic methods can also increase longevity by modifying these pathways, which suggests that it might be possible to develop drugs that can increase lifespan without reducing calorie intake.

Mice, humans and other creatures respond to prolonged fasting through a number of adaptive changes that include mobilizing and burning fatty acids. The liver has an important role in this response, secreting a hormone called fibroblast growth factor-21 (FGF21) that coordinates these processes among tissues. Previous experiments on transgenic mice with high levels of this hormone have shown that it suppresses the activity of growth hormone and reduces the production of insulin-like growth factor, which prevents growth and can lead to hibernation-like behavior.

Here Zhang et al. compare groups of wild-type mice and transgenic mice with high levels of FGF21. They find that the transgenic mice have a longer median survival time than wild-type mice (38 months vs 28 months), and that the transgenic female mice on average live for 4 months longer than their male counterparts. However, unlike in other examples of increased longevity, they find that decreased food intake is not required. Instead, they find that transgenic mice eat more food than wild-type mice, yet remain profoundly insulin-sensitive. The results suggest that the longer survival times are caused by a reduction in the production of insulin-like growth factor, but they also suggest that the mechanism responsible for the increased longevity is independent of the three pathways that are usually associated with such increases. Further research is needed to understand this mechanism in greater detail and could, perhaps, pave the way for the use of FGF21-based hormone therapy to extend lifespan without the need for a low-calorie diet.

**DOI:** http://dx.doi.org/10.7554/eLife.00065.002

## Introduction

Caloric restriction without malnutrition is a proven means of inhibiting aging in species ranging from worms to nonhuman primates (Masoro, 2005; Bishop and Guarente, 2007; Kenyon, 2010). The effect of caloric restriction on longevity appears to be mediated by multiple nutrient-sensing pathways including those involving insulin and insulin-like growth factor (IGF-1), target of rapamycin (TOR), AMP kinase and sirtuins. Pharmacologic and/or genetic manipulation of these pathways increases longevity to varying degrees, suggesting the feasibility of drugs that increase lifespan in the absence of caloric restriction (Bishop and Guarente, 2007; Kenyon, 2010; Barzilai et al., 2012).

Fibroblast growth factor-21 (FGF21) is an atypical FGF that functions as an endocrine hormone (Potthoff et al., 2012). In mice, FGF21 is strongly induced in liver in response to prolonged fasts through a peroxisome proliferator-activated receptor α-dependent mechanism. FGF21 in turn elicits diverse aspects of the adaptive starvation response. Among these, FGF21 increases insulin sensitivity and causes a corresponding decrease in basal insulin concentrations; FGF21 increases hepatic fatty acid oxidation, ketogenesis and gluconeogenesis; and, FGF21 sensitizes mice to torpor, a hibernation-like state of reduced body temperature and physical activity (Potthoff et al., 2012). FGF21 also blocks somatic growth by causing GH resistance, a phenomenon associated with starvation. Transgenic (Tg) mice overexpressing FGF21 are markedly smaller than wild-type mice and have a corresponding decrease in circulating IGF-1 concentrations despite having elevated growth hormone (GH) levels (Inagaki et al., 2008). Conversely, FGF21-knockout mice grow more than wild-type mice under conditions of nutrient deprivation (Kubicky et al., 2012). In liver, FGF21 inhibits the GH signaling pathway by blocking JAK2-mediated phosphorylation and nuclear translocation of the transcription factor, STAT5. This suppresses the transcription of *Igf1* and other GH/STAT5-regulated genes (Inagaki, et al., 2008). Thus, FGF21-mediated repression of the GH/IGF-1 axis provides a mechanism for blocking growth and conserving energy under starvation conditions.

Dwarf mice, including the pituitary loss-of-function Ames and Snell strains and GH receptor/GH binding protein-knockout mice, are the longest living mouse mutants discovered to date, living up to ∼70% longer than their wild-type counterparts (Liang et al., 2003; Bartke and Brown-Borg, 2004; Brown-Borg and Bartke, 2012). Interestingly, FGF21-Tg mice share a number of phenotypes with these long-lived mice including small size, enhanced insulin sensitivity and a blunted GH/IGF-1 signaling axis. In this report, using FGF21-Tg mice, we examine the consequences of chronic FGF21 exposure on lifespan.

## Results

We previously described FGF21-Tg mice in which the FGF21 transgene is selectively expressed in hepatocytes under the control of the apoE promoter (Inagaki et al., 2007, 2008). Circulating concentrations of FGF21 are ∼5–10-fold higher in the FGF21-Tg mice than under fasted conditions. Younger FGF21-Tg mice (<8-month-old) have significant decreases in serum insulin, IGF-1, glucose, triglycerides and cholesterol and in hepatic triglyceride levels (Inagaki et al., 2007, 2008). Similar effects on insulin, glucose, triglycerides and cholesterol levels were seen in FGF21-Tg mice in which the FGF21 transgene was under the control of the albumin promoter (Kharitonenkov et al., 2005).

**Figure 1.**
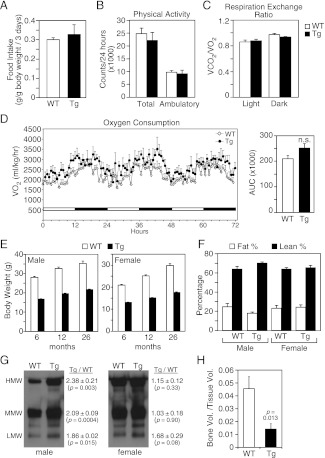
Metabolic parameters in aging wild-type and FGF21-transgenic mice. (**A**) Food intake, (**B**) physical activity, including total horizontal and ambulatory activity, (**C**) respiratory exchange ratio (VCO_2_/VO_2_) and (**D**) oxygen consumption (VO_2_) data for 30-month-old male wild-type (WT) and FGF21-transgenic (Tg) mice (n=6/group) housed singly in metabolic cages. For (**D**), the area under the curve (AUC) data are shown in the right panel. (**E**) Body weights of male and female mice measured at 6, 12 and 26 months. Measurements were done on all surviving mice in the cohorts (n=27–54/group). (**F**) Fat and lean mass percentages were measured in 26-month-old mice (n=5–6/group). (**G**) Adiponectin oligomer forms, including high molecular weight (HMW), medium molecular weight (MMW) and low molecular weight (LMW) forms, were measured in plasma from 26- to 27-month-old male and female WT and FGF21-Tg mice. Representative western blots using plasma from single animals are shown together with the ratios of the different adiponectin forms in FGF21-Tg and WT plasma (n=4/group). (**H**) Quantification of trabecular bone volume by μCT analysis using tibiae from 33- to 35-month-old male WT and FGF21-Tg mice (n=4–5/group). All data are presented as the mean ± SEM. **DOI:** http://dx.doi.org/10.7554/eLife.00065.003

We examined whether these and additional metabolic parameters were altered in groups of older (26–27-month-old) wild-type and FGF21-Tg mice. There were no differences in food intake, physical activity, oxygen consumption or respiratory exchange ratio (Figure 1A–D). Although both male and female FGF21-Tg mice weighed less than their wild-type littermates, there were no differences in their percent fat and lean mass (Figure 1E,F). Accordingly, plasma leptin levels were not significantly changed in FGF21-Tg mice (Table 1). Plasma adiponectin concentrations were significantly higher in male FGF21-Tg mice (Table 1), with increases in both the monomeric and more active oligomeric forms (Figure 1G). There was no significant change in either the total amount or the different forms of adiponectin in females (Table 1, Figure 1G). Plasma ketone body levels were significantly higher in female but not male FGF21-Tg mice (Table 1). Hepatic triglyeride concentrations were lower in female but not male FGF21-Tg mice (Table 1). Plasma and hepatic cholesterol concentrations were unchanged in FGF21-Tg mice (Table 1). As expected (Wei et al., 2012), FGF21-Tg mice had reduced bone mass (Figure 1H).

**Table 1.** Plasma and hepatic parameters **DOI:** http://dx.doi.org/10.7554/eLife.00065.004

	Male			Female		
	WT	Tg	p	WT	Tg	p
Plasma						
IGF-1* (ng/mL)	381.52±42.31	250.1±13.76	0.03	427.94±56.35	171.84±11.71	0.009
Insulin* (ng/mL)	0.90±0.13	0.45±0.09	0.04	0.63±0.12	0.34±0.04	0.08
Glucose* (mg/dL)	155±9.21	111.6±8.03	0.008	181.75±19.77	121.4±7.50	0.048
Ketone bodies* (μM)	118.38±30.51	221.02±38.10	0.07	140.61±17.46	294.56±48.10	0.03
Adiponectin (μg/mL)	9.43±1.65	23.42±2.65	0.006	24.13±2.82	31.71±7.97	0.42
Leptin (ng/mL)	3.20±1.02	4.14±1.09	0.55	4.80±1.82	7.46±2.89	0.47
Triglycerides (mg/dL)	75.14±9.23	51.82±8.63	0.11	50.28±10.31	21.79±6.23	0.06
Cholesterol (mg/dL)	83.20±10.18	81.45±2.32	0.88	79.82±9.44	70.56±13.58	0.60
Liver						
Triglycerides (mg/g)	11.62±2.23	6.67±0.75	0.11	22.42±3.75	10.33±0.68	0.04
Cholesterol (mg/g)	4.29±0.50	4.07±0.20	0.71	4.52±0.22	4.54±0.13	0.94

Measurements were made using 26–27-month-old mice (n=4–5 mice/group).

* 4 hr fasting data.

**Figure 2.**
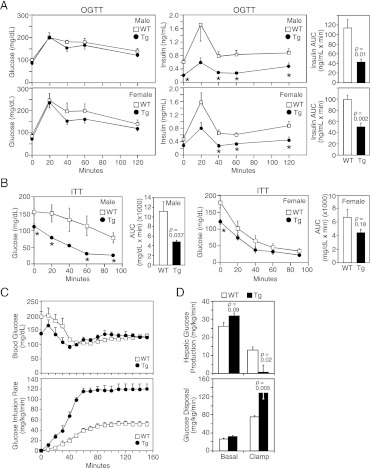
FGF21-transgenic mice have increased insulin sensitivity. 26–27-month-old wild-type (WT) and FGF21-transgenic (Tg) male and female mice (n=5/group) were subjected to (**A**) oral glucose tolerance tests or (**B**) insulin tolerance tests. Plasma glucose and insulin levels were measured as indicated. Quantification of the area under the curve (AUC) is shown in the panels to the right. (**C, D**) Hyperinsulinemic-euglycemic clamp studies were performed on 6- to 8-month-old male WT and FGF21-Tg mice (n=6/group). Insulin was infused at 2 mU/kg/min and (**C**) blood glucose (upper panel) was clamped at 120 mg/dL during the steady-state period (t=110–150 min) using a variable glucose infusion rate (lower panel). The glucose infusion rate in FGF21-Tg mice is statistically different (p<0.05) from WT mice at all points after t=0 min. (**D**) Rates of hepatic glucose production (upper panel) and whole-body glucose disposal (lower panel) in WT (n=6) and FGF21-Tg (n=3) mice during the basal and steady-state periods of the clamp. **DOI:** http://dx.doi.org/10.7554/eLife.00065.005

We next examined glucose homeostasis in older mice. Under 4 hr fasted conditions, plasma glucose concentrations were lower in both male and female FGF21-Tg mice, while plasma insulin concentrations were significantly lower in male FGF21-Tg mice (Table 1). Plasma IGF-1 concentrations were also lower in both male and female FGF21-Tg mice, with the decrease particularly striking in females (Table 1). In oral glucose tolerance tests done in mice fasted for 16 hr, there was no difference in glucose excursion between wild-type and FGF21-Tg mice, but the FGF21-Tg mice had significantly lower insulin levels in response to the glucose challenge (Figure 2A). In insulin tolerance tests done in male mice fasted for 4 hr, plasma glucose concentrations decreased more in FGF21-Tg mice than in wild-type mice (Figure 2B).

These data suggested that the FGF21-Tg mice have increased insulin sensitivity. To address this directly, hyperinsulinemic-euglycemic clamp experiments were performed. The exogenous glucose infusion rate required to maintain euglycemia under clamp conditions was markedly higher in FGF21-Tg mice than in wild-type mice, demonstrating enhanced whole-body insulin sensitivity (Figure 2C). Glucose tracer kinetic analysis revealed that insulin-stimulated suppression of hepatic glucose production and activation of whole-body glucose disposal were significantly greater in FGF21-Tg mice than in wild-type mice (Figure 2D). Clamp insulin levels were similar between wild-type and FGF21-Tg mice (7.0±2.0 and 8.3±2.3 ng/mL, respectively; not significantly different). Together, these data demonstrate that glucose tolerance and whole-body insulin sensitivity are dramatically increased in FGF21-Tg mice.

**Figure 3.**
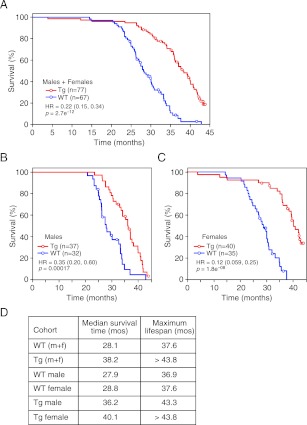
FGF21 extends lifespan. (**A–C**) Kaplan–Meyer survival curves for wild-type (WT) and FGF21-transgenic (Tg) mice are shown. (**A**) Combined male and female data; (**B**) male data; (**C**) female data. (**D**) Median survival time (at 50th percentile) and maximum lifespan (at 95th percentile) for each cohort. Hazard ratios (HR) and 95% confidence intervals are shown for Tg vs WT mice. **DOI:** http://dx.doi.org/10.7554/eLife.00065.006

Given the effects of long-term FGF21 exposure on carbohydrate and lipid parameters, especially insulin and IGF-1 concentrations, we measured the lifespan of FGF21-Tg mice. Longevity was significantly extended in FGF21-Tg mice compared to wild-type littermates (hazard ratio=0.22 [0.15, 0.34], p=2.7e^−12^), with a 36% increase in the median survival time for FGF21-Tg mice (Figure 3A). Although there was no difference in longevity between male and female wild-type mice, the difference in lifespan between male and female FGF21-Tg mice was statistically significant (hazard ratio=2.42 [1.37, 4.26], p=0.0023) (Figure 3B,C). Cox proportional-hazards regression analysis shows that FGF21 reduced the risk of death by 65% in males (hazard ratio=0.35 [0.20, 0.60], p=0.00017) and 88% in females (hazard ratio=0.12 [0.059, 0.25], p=1.8e^−08^) (Figure 3B,C). Overall, there was a strong interaction between the presence of the FGF21 transgene and sex (p=0.01). Notably, at the time of this analysis >30% of the age-matched female FGF21-Tg mice were still alive at 44 months of age.

**Figure 4.**
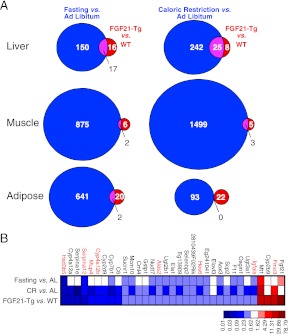
Genes regulated by FGF21 and caloric restriction overlap in liver. (**A**) Venn diagrams showing overlap of genes significantly regulated in liver, muscle and adipose tissue of FGF21-transgenic (Tg) vs wild-type (WT) mice (FDR<0.10, >twofold regulation) compared to the same gene expression analysis in calorically restricted (CR) vs ad libitum (AL) or fasted vs AL mice. (**B**) Heat map of genes significantly regulated in liver of FGF21-Tg vs WT (FDR<0.10, >twofold regulation) compared to expression of the same liver gene set regulated by fasting or CR. Microarray analysis was performed using liver, epididymal white adipose tissue and gastrocnemius muscle from wild-type and FGF21-Tg male mice and male C57BL/6J mice subjected to 60% caloric restriction for 2 weeks or a 24 hr fast. All mice used in these studies were 3 months old at the end of the study. **DOI:** http://dx.doi.org/10.7554/eLife.00065.007

Since FGF21 is induced by fasting and elicits diverse aspects of the adaptive starvation response, we examined whether chronic FGF21 exposure mimics nutrient deprivation with respect to changes in gene expression. Comprehensive transcriptome analysis was performed by microarray using RNA from liver, gastrocnemius muscle and epididymal white adipose tissue of wild-type and FGF21-Tg mice and mice subjected to either caloric restriction or a 24 hr fast. Using a false discovery rate <0.10 and fold change >2 as criteria, we found that expression of 33, 8 and 22 genes was changed in liver, muscle and adipose of FGF21-Tg mice, respectively (Figure 4A). Many more genes were regulated by caloric restriction or fasting than by the FGF21 transgene in all three tissues. As expected, *Fgf21* was strongly induced in liver by fasting. Surprisingly, however, *Fgf21* was not induced by the caloric restriction regimen (Figure 4B). Likewise, there was no increase in plasma FGF21 concentrations in response to caloric restriction (data not shown). While the molecular basis for this differential regulation of *Fgf21* by fasting and caloric restriction is not yet known, these data indicate that FGF21 is not an endogenous mediator of the caloric restriction response. Notably, 30 of the 33 genes with changed expression in liver of FGF21-Tg mice were also regulated by caloric restriction, while 20 of these genes were regulated by fasting (Figure 4B). Eight of the genes with altered expression in liver of FGF21-Tg mice (highlighted in red in Figure 4B; see Discussion) are similarly regulated in long-lived dwarf mice (Swindell, 2007). In contrast, there was little overlap in genes regulated by FGF21 and either caloric restriction or fasting in muscle or adipose. These data suggest that FGF21 may extend lifespan by regulating a small subset of genes also regulated by caloric restriction in liver.

**Figure 5.**
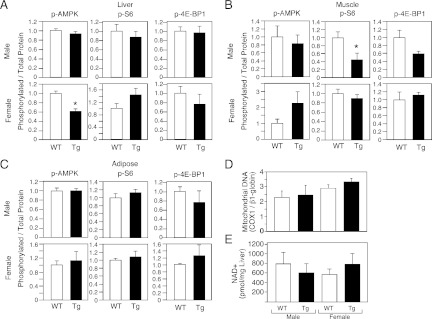
Evaluation of markers of AMP kinase, mTOR and sirtuin pathway activity in FGF21-Tg mice. Phosphorylated levels of AMP kinase, S6, and 4E-BP1 in (**A**) liver, (**B**) gastrocnemius muscle, and (**C**) epididymal white adipose tissue; (**D**) mitochondrial DNA content and (**E**) NAD+ concentrations in liver of 26_28-month-old male and female wild-type (WT) and FGF21-transgenic (Tg) mice (n=4/group except for female adipose tissue, where n=2/group; all data are presented as the mean ± SEM; *p<0.05). **DOI:** http://dx.doi.org/10.7554/eLife.00065.008

Increases in AMP kinase and sirtuin activity and decreases in mTOR activity are associated with increased longevity (Bishop and Guarente, 2007; Kenyon, 2010). To begin to assess whether these pathways are affected by FGF21, we measured phosphorylated and total levels of AMP kinase and the mTOR targets S6 and 4E-BP1in liver, muscle and adipose tissue of male and female wild-type and FGF21-Tg mice. We also determined mitochondrial DNA content in liver as a downstream measure of AMP kinase activity. Phospho-AMP kinase levels were not increased in tissues from FGF21-Tg mice (Figure 5A–C). Consistent with these data, mitochondrial DNA content was unchanged in liver (Figure 5D). While Phospho-S6 and phospho-4E-BP1 levels were decreased in muscle of male FGF21-Tg mice (Figure 5B), they were unchanged in muscle of longer-lived FGF21-Tg females or in liver or adipose from either sex (Figure 5A–C). We also did not observe increases in NAD+ concentrations (Figure 5E) or the mRNA levels of Sirtuins 1–7 (data not shown) in liver of FGF21-Tg mice, suggesting that sirtuin activity is unlikely to be increased. Taken together, these data suggest that FGF21 may increase longevity through a mechanism independent of the AMP kinase, mTOR and sirtuin pathways.

## Discussion

In this report, we demonstrate that chronic exposure of mice to the starvation hormone, FGF21, increases median survival time by ∼30% and ∼40% in males and females, respectively, without decreasing food intake. The increase in lifespan extension is comparable to that achieved by caloric restriction (Turturro et al., 1999). While the FGF21-mediated increase in longevity is less than the 50–70% increase seen in pituitary loss-of-function Ames mice and GH receptor/GH binding protein-knockout mice (Brown-Borg et al., 1996; Coschigano et al., 2000), it is similar to that seen in other loss-of-function dwarf models including hypopituitary Snell mice (Flurkey et al., 2002) and mice lacking pregnancy-associated plasma protein-A (PAPP-A), a protease that increases IGF-1 activity (Conover et al., 2010). The FGF21-Tg mice share other phenotypic similarities with long-lived dwarf mice including small size, reduced circulating insulin and IGF-1 concentrations, increased circulating adiponectin levels and female infertility. Like the Ames and Snell dwarf mice, female FGF21-Tg mice live longer than males (Liang et al., 2003; Brown-Borg and Bartke, 2012). These similarities together with our previous finding that FGF21-Tg mice are GH resistant (Inagaki et al., 2008) suggest that FGF21 may increase lifespan by inhibiting the GH/IGF-1 signaling pathway. Because FGF21 also regulates other pathways that impact metabolism, it is not surprising that some of the effects that are seen in dwarf mice, such as increased adiposity in GH receptor-knockout mice (Masternak et al., 2012), are not recapitulated in FGF21-Tg mice.

In microarray studies, FGF21 modulated more genes in liver than in either muscle or adipose. Interestingly, nearly all of these hepatic genes (30 of 33) were similarly regulated by caloric restriction, a proven method of extending lifespan in mice and other species (Masoro, 2005; Bishop and Guarente, 2007; Kenyon, 2010). In contrast, there was little or no overlap in the genes regulated by FGF21 and caloric restriction in muscle or adipose. A remarkable result of our studies is that FGF21 increased longevity to a similar extent as caloric restriction while regulating a much smaller set of genes in liver. These data suggest that FGF21 may extend lifespan by acting as a selective caloric restriction mimetic in liver. An important caveat, however, is that there may be changes in other tissues or post-transcriptional changes that were missed in this analysis. We note that caloric restriction did not increase circulating FGF21 levels, indicating that endogenous FGF21 does not mediate the longevity effects of caloric restriction.

Using microarray analysis, Swindell (2007) previously defined a set of 43 candidate longevity genes based upon their similar regulation in different dwarf mouse strains or between dwarf strains and caloric restriction. Eight of these genes (*Fmo3*, *Igfals*, *Hes6*, *Alas2*, *Cyp4a12b*, *Mup4*, *Serpina12*, *Hsd3b5*) are among those co-regulated by FGF21 and caloric restriction in liver in our current study, and four others (*Igf1*, *Igfbp1*, *Socs2* and *Mup3*) were previously shown by more sensitive quantitative real-time PCR to be regulated in liver of FGF21-Tg mice (Inagaki et al., 2008). Strikingly, 26 of the 33 genes regulated by FGF21 in liver were previously shown to be regulated in a similar manner in mice lacking STAT5 activity (Barclay et al., 2011). Thus, FGF21 may increase longevity by suppressing the GH signaling axis in liver in a manner similar to that of caloric restriction. Interestingly, GH induces FGF21 in liver through a STAT5-dependent mechanism (Barclay et al., 2011; Chen et al., 2011; Yu et al., 2012). This regulatory relationship between GH and FGF21 is likely to be important in controlling GH activity and coordinating energy homeostasis during starvation.

The ability to increase lifespan adds to a growing list of other beneficial effects that pharmacologic administration of FGF21 has in mammals, including insulin sensitization, normalization of glycemia, and reduced body weight in obese animals (Potthoff et al., 2012). These attributes have made FGF21 an intriguing therapeutic target. In contrast to these positive effects, however, we previously showed that younger (<8-month-old) FGF21-Tg mice and adult mice administered recombinant FGF21 for 14 days have reduced bone mass (Wei et al., 2012). In our current study, similar reductions in bone mass were seen in older mice. The FGF21-dependent decrease in bone mass is caused in part by an increase in the differentiation of marrow adipocytes and corresponding decrease in osteoblast differentiation (Wei et al., 2012). In addition, FGF21 inhibits GH action directly at the growth plate (Wu et al., 2012), and long-lived PAPP-A-knockout mice have a decrease in bone mineral density (Tanner et al., 2008), suggesting that changes in GH/IGF-1 signaling are also likely to be involved in FGF21-induced decreases in bone mass. While it remains to be determined whether FGF21 causes bone loss in humans, it is likely that this adverse effect will have to be overcome if FGF21 is to be used clinically to combat aging.

In summary, we show that chronic exposure to FGF21, a naturally-occurring hormone and potent insulin sensitizer, markedly extends lifespan in mice through a mechanism that may involve suppression of the GH/IGF-1 signaling axis in liver. Regarding other hormones that influence lifespan, we note that the extracellular domain of Klotho, a membrane protein that regulates phosphate metabolism and serves as a co-receptor for FGF23 but not FGF21, can be cleaved to yield a circulating peptide that blocks insulin and IGF-1 signaling. This hormonal activity may contribute to Klotho’s lifespan-extending effect in mice (Kurosu et al., 2005). However, unlike FGF21, Klotho causes insulin resistance, which may limit its utility as a therapeutic agent. We conclude that FGF21 could potentially be used as a hormone therapy to extend lifespan in mammals.

## Materials and methods

### Animal experiments

FGF21-Tg mice and wild-type littermates (Inagaki et al., 2007) were maintained on C57Bl/6J background in a specific-pathogen-free facility with 12:12 light:dark cycle, and fed 2916 global diet (Harlan). Littermates were housed in the same cage with a maximum number of five mice per cage. Cages were changed every week and nesting material was provided. Animals were sacrificed if they had wounds from fighting, developed severe dermatitis, tumors or other signs of morbidity. Caloric restriction experiments were performed with C57Bl/6J mice. Eight-week-old male mice were individually caged and fed ad libitum. Food intake was measured for 1 week. For the following 2 weeks, each mouse was fed at 4 p.m. every day with food equal to 40% of the daily consumption in the first week. Body weight and total body fat composition were measured 1 hr before feeding. The control group received food ad libitum. At the end of the study, mice from the caloric restricted group, ad libitum fed group, and a 24 hr fast group were sacrificed between 2 and 4 p.m. and blood and tissues were collected for analysis. All animal experiments were approved by the Institutional Animal Care and Research Advisory Committee at the University of Texas Southwestern Medical Center.

### Metabolic cage analysis

Mice were individually housed in CLAMS system metabolic cages and were allowed to acclimate for 1 day. Oxygen consumption, CO2 production, activity and food intake data were collected for 3 days as described (Kalaany et al., 2005).

### Body composition and bone analyses

Body fat and lean mass were measured using an EchoMRI-100 (Echo Medical Systems, LLC). Bone mass was determined by μCT using tibiae as described (Wei et al., 2012).

### Plasma analyses

Blood was collected into EDTA-coated tubes (Starstedt, Newton, NC). Plasma was separated by centrifugation and assayed using commercially-available kits for IGF-1 (Alpco), total cholesterol (Thermo Scientific), triglycerides (Thermo Scientific), total ketone bodies (Wako Chemicals), leptin and total adiponectin (Millipore). The oligomeric forms of adiponectin were measured essentially as described (Waki et al., 2003). Briefly, 0.5 μL of plasma from individual mice was subjected to SDS-PAGE under non-reducing and non-heat-denaturing conditions and western blot analysis performed using anti-adiponection mouse polyclonal antiserum (a gift from Philipp Scherer).

### Glucose and insulin tolerance tests

Glucose tolerance tests were performed on overnight fasted mice. Twenty percent D-glucose (Sigma) (1 g/kg body weight) was administered by oral gavage. At 0, 20, 40, 60, and 120 min after administration, blood was collected by tail vein bleeding. Glucose levels were measured by enzymatic assay (Wako Chemicals). Insulin levels were measured by ELISA (Crystal Chem). Insulin tolerance tests were performed on mice after a 4 hr fast. 0.75 U/kg of human insulin (Sigma) was intraperitoneally injected into mice. At 0, 20, 40, 60, and 90 min after injection, tail vein blood was drawn and glucose levels measured using a One Touch Ultra glucometer (Life Scan).

### Hyperinsulinemic-euglycemic clamp analysis

Insulin sensitivity was evaluated in 6- to 8-month-old male WT and FGF21-Tg mice (n=6/group) by performing hyperinsulinemic-euglycemic clamp experiments as described (Ayala et al., 2006; Berglund et al., 2009). Briefly, 5 days prior to the clamp studies, infusion catheters were implanted in the right jugular vein under isofluorane anesthesia, tunneled subcutaneously, and exteriorized at the back of the neck. On the morning of the experiment, mice were transferred to sterile cages with bedding and water to begin a 4 hr fast. At t=−90 min, water was removed and a primed continuous infusion of HPLC-purified [3-^3^H]glucose (5 μCi bolus + 0.05 μCi/min) was started for the assessment of glucose turnover. Blood samples were obtained from the cut tail at t=−15 and −5 min for the measurement of basal blood glucose (AlphaTRAK glucometer, Abbott, Chicago, IL). At t=0 min, a primed continuous infusion of insulin (6 mU + 2.0mU/kg/min; Humulin) was started to induce hyperinsulinemia, and the [3-^3^H]glucose infusion rate was doubled (0.1 μCi/min) to minimize alterations in specific activity. Blood glucose was measured every 10 min thereafter, while 50% dextrose was infused at a variable rate to maintain target glycemic levels at 120 mg/dL. Glucose turnover was calculated during the basal (t=−15 to −5 min) and steady-state (t=110–150 min) periods of the clamp using Steele's steady-state equation (Steele et al., 1956).

### Liver lipid measurements

Liver triglyceride and cholesterol were extracted from 0.1 g of liver tissue by the Folch method as described (Kalaany et al., 2005). Total cholesterol or triglycerides were measured by colorimetric enzymatic assays (Thermo Scientific).

### Western blot analysis

Liver and adipose tissue proteins were extracted as described (Inagaki et al., 2008; Dutchak et al., 2012). Gastrocnemius protein was extracted in 140 mM NaCl, 10 mM Tris-HCl (pH 8.1), 1 mM CaCl_2_, 1 mM MgCl_2_, 10% glycerol, 1% NP-40 with Complete protease inhibitor cocktail (Roche diagnostics). 30 mg of whole liver lysate was resolved on a SDS-polyacrylamide gels and electrotransferred to a PVDF membrane (Amersham). The membrane was then hybridized with antibodies against total AMPK, phospho-AMPK (T172), total S6 and phospho-S6 (S240/244) (Cell Signaling) and total and phospho-4E-BP1 (T70) (Cell Signaling). Protein was detected by chemiluminescence (ECL kit, Amersham). Results were quantified by densitometry using ImageJ Software (NIH).

### Mitochondrial DNA content

Genomic DNA was isolated from liver using a DNeasy Blood and Tissue kit (Qiagen). The mitochondrial gene COX-1 was measured by quantitative real-time PCR with β-globin used as a control as described (Sahin et al., 2011).

### NAD+ measurements

Frozen liver tissues were extracted and NAD+ levels were measured using HPLC as described (Ramsey et al., 2009).

### Survival analysis

Survival time was calculated from the date of birth until the last date of the observation. Mice that were still alive at the end of study or sacrificed during the study were censored at the last date of the observation. Survival curves were estimated using the Kaplan–Meier curves (Kaplan and Meier, 1958) and were compared using the log-rank test. The univariate and multivariate survival analyses were performed using Cox proportional-hazards analysis (Collett, 2003). Median survival time was calculated as the shortest survival time for which the survivor function is ≤0.5. Maximum lifespan was calculated as the shortest survival time for which the survivor function is ≤0.95.

### Microarray data analysis

RNA was prepared from liver, epididymal white adipose and gastrocnemius muscle from 3-month-old wild-type and FGF21-Tg mice. RNA was reverse transcribed into cRNA and biotin-UTP labeled using the Illumina TotalPrep RNA Amplification Kit (Ambion) and hybridized to the Illumina mouseRefseq-8v2 Expression BeadChips. Hybridizations were done in triplicate with each replicate containing cRNA from 1 to 3 different mice. Image data were converted into unnormalized Sample Probe Profiles using the Illumina BeadStudio software and raw expression data were processed using the Model-Based Background Correction method (Xie et al., 2009). All gene expression values were log2 transformed and quantile normalized. Significance Analysis of Microarrays (Tusher et al., 2001) was used to identify differentially expressed genes among different groups using a false discovery rate of less than 10% and fold-change >2 as criteria.

### Statistical analyses

Results were analyzed by a Student’s unpaired t-test using GraphPad Prism (GraphPad Software, Inc.). All data are presented as the mean ± SEM.

### Accession numbers

The microarray data have been deposited in the NCBI Gene Expression Omnibus with accession number GSE39313.

## Additional files

### Major datasets

The following datasets were generated

YuRuth T, 2012, The role of FGF21 in aging and aging-related diseases, ; http://www.ncbi.nlm.nih.gov/geo/query/acc.cgi?acc=GSE39313, In the public domain at GEO: http://www.ncbi.nlm.nih.gov/geo/

**Reporting standards:** Microarray data were deposited in the NCBI Gene Expression Omnibus (GEO) repository
